# Precision stratified treatment of primary hyperparathyroidism based on multilevel emerging biomarkers

**DOI:** 10.3389/fendo.2025.1688399

**Published:** 2025-10-24

**Authors:** Xi Zhu, Qi Zhang, Linghui Wang, Shuting Xu, Enmei Gong, Bin Zhou, Yong Wu, Zhouting Li, Yanjie Zhao, Shuhui Li, Feng Cheng, Lei Zhu

**Affiliations:** ^1^ Department of Head and Neck Surgery, the Fifth Hospital Affiliated to Wenzhou Medical University, Lishui Central Hospital, Lishui, Zhejiang, China; ^2^ Hangzhou Normal University, Hangzhou, Zhejiang, China; ^3^ Zhejiang Chinese Medical University, Hangzhou, Zhejiang, China; ^4^ Hangzhou Medical College, Hangzhou, Zhejiang, China; ^5^ Department of Pediatric Surgery, the Fifth Hospital Affiliated to Wenzhou Medical University, Lishui Central Hospital, Lishui, Zhejiang, China

**Keywords:** primary hyperparathyroidism, precision medicine, multilevel biomarkers, stratified therapy, multidisciplinary team

## Abstract

Primary hyperparathyroidism (PHPT) is a clinically heterogeneous endocrine disorder whose management has historically been guided by biochemical profiles and symptomatic presentation. However, the limitations of conventional diagnostic and therapeutic strategies—including poor sensitivity in detecting subclinical organ damage and an inability to predict disease progression—have underscored the need for a more nuanced, biomarker-driven approach. Recent advances in multi-omics technologies, functional imaging, and artificial intelligence have enabled the emergence of precision medicine paradigms for PHPT. This review synthesizes evidence on the role of multilevel biomarkers—spanning genetic, epigenetic, non-coding RNA, metabolic, and imaging domains—in refining risk stratification and guiding individualized treatment. We further propose an integrated framework that combines dynamic biomarker profiling with multidisciplinary team (MDT)-based decision-making to facilitate personalized intervention pathways—from surgical planning to long-term surveillance. Despite promising developments, clinical translation remains challenged by the lack of standardized biomarker validation and integrative analytical platforms. Future efforts should prioritize collaborative networks and large-scale prospective studies to establish evidence-based guidelines for implementing precision medicine in PHPT.

## 1 Introduction

### 1.1 Disease definition and pathophysiological basis

Hyperparathyroidism (HPT) can be classified into three major clinical types based on pathophysiological features: primary, secondary and tertiary, and its etiological spectrum is significantly heterogeneous. PHPT is characterized by autonomous secretion of parathyroid hormone (PTH) by parathyroid tissues, and about 80-85% of cases originate from monoclonal parathyroid adenomas (1), the molecular mechanism of which involves cyclin D1 (CCND1/PRAD1) overexpression and the development of Wnt/β-cyclin. aberrant activation of the Wnt/β-linker pathway (2), whereas the genetic etiology is most commonly seen in multiple endocrine neoplasia syndromes (MEN1, MEN2A, MEN4) or parathyroid-jaw tumor syndrome (HPT-JT), which are closely associated with germline mutations in genes such as *MEN1* and *CDC73* (3, 4). In addition, 15% to 20% have multiple parathyroid gland hyperplasias or adenomas (5), which are often associated with abnormalities in calcium regulatory sites due to mutations in calcium-sensing receptor (CaSR) signaling pathway genes (*CASR, GNA11, AP2S1*), and environmental factors, such as a history of cervical radiation or prolonged lithium treatment, can contribute to the development of the disease by interfering with the function of the CaSR (2). Approximately 1% are parathyroid carcinomas, associated with inactivation of the *CDC73/HRPT2* double allele, *RET* mutations, and in some cases HPT-JT or MEN1 syndrome (4). The nature of secondary hyperparathyroidism (SHPT) is a compensatory response of the parathyroid glands to chronic pathological stimuli, with core triggers including chronic kidney disease (CKD)-related phosphorus retention, active vitamin D deficiency and fibroblast growth factor 23 (FGF23). Growth Factor 23 (FGF23) levels, which together lead to hypocalcaemia and sustained activation of PTH secretion (6–8). SHPT can also be induced by impaired calcium absorption due to gastrointestinal disorders (e.g., celiac disease, post-bariatric surgery) and by pharmacological (lithium, diuretics) disturbances of calcium homeostasis (9). Notably, prolonged SHPT can progress to tertiary hyperparathyroidism (THPT), which is characterized by monoclonal amplification of parathyroid tissue in response to persistent stimulation, resulting in the formation of autocrine nodules or adenomas (8, 10). At the molecular level, the pathogenesis of THPT involves significant down-regulation of calcium-sensitive receptor (CaSR) and vitamin D receptor (VDR) expression, and whole-exome sequencing studies have further revealed that mutations in genes such as PRKDC and TBX20 drive malignant transformation by disrupting cell cycle regulation and DNA repair pathways (11). Such lesions are most common in CKD patients on long-term dialysis or after renal transplantation, suggesting that the synergistic effects of chronic metabolic disorders and genetic damage are critical in disease progression (2, 8, 10).

### 1.2 The PHPT landscape: epidemiology, clinical subtypes, and core biochemical profile

PHPT is a common endocrine disorder whose epidemiological profile shows significant gender, age and ethnic differences. Females account for 75% of cases, especially postmenopausal women, and may be associated with the high expression of estrogen receptor beta (ERβ) in parathyroid adenomas and its pro-apoptotic effects (12). In terms of age distribution, it is rare in people under 40 years of age (about 5% of cases), 65% of patients over 65 years of age, and 85% over 50 years of age, whereas the incidence decreases over 80 years of age and the sex ratio is balanced. In terms of racial differences, the prevalence is higher in Asian and black groups, and Asian patients have an earlier age of onset. Environmental factors such as exposure to endocrine disruptors such as polychlorinated biphenyls (PCBs) may increase the risk, while misdiagnosis of Familial Hypocalciuric Hypercalcemia (FHH) may also influence the epidemiological data (12). The prevalence varies significantly globally, ranging from 17-946.6/100,000/year, with countries where routine biochemical screening is widely available, such as the United States, having prevalence rates of 48.3-504/100,000/year, up to 233/100,000 females and 85/100,000 males in the general population in the U.S., and with a significantly higher prevalence rate in black females aged 70–79 years (1,409/100,000) is significantly higher than in whites (1,110/100,000) (13). The overall prevalence in Scotland 2007–2018 was 0.84 per cent (1.18 per cent for women and 0.48 per cent for men), and about 0.4 per cent in places such as South Korea and Bahrain, while referral hospitals could be up to 1.3 per cent. In special populations, the prevalence is about 1% in pregnant women and up to 1.5% in the elderly (>80 years). The prevalence of the asymptomatic form (normal blood calcium PHPT) ranges from 0.18% to 3.1% and is often underestimated (12).

The clinical classification criteria for PHPT are mainly based on the presence or absence of symptoms and changes in blood calcium levels and PTH levels. The classification system of PHPT is based on multidimensional disease characteristics. The primary basis for classification is the patient’s clinical presentation. Based on the presence of target organ damage directly caused by hypercalcemia or PTH overproduction, PHPT can be clearly differentiated into symptomatic PHPT and asymptomatic PHPT (14–16). The clinical manifestations of symptomatic PHPT encompass multi-system involvement, and neuropsychiatric symptoms are seen in about 25% of patients, manifesting as depression, anxiety, cognitive impairment and insomnia, and progressing to acute psychosis (e.g., hallucinations, delusions) in severe cases, and the mechanism of which may be related to hypercalcaemia interfering with the metabolism of monoamine neurotransmitters and the activation of pro-inflammatory cytokines (TNFα, IL-17) (17). Digestive system involvement is characterized by pancreatitis, gastric ulcers (prevalent in patients with MEN1 syndrome), constipation and cholelithiasis, with hypercalcaemia leading to pancreatic ductal obstruction by promoting gastric acid secretion, inhibiting intestinal smooth muscle contraction and inducing pancreatic calcification (18, 19). Urological damage is typified by renal calculi (incidence 5-55%), renal calcinosis (incidence ~10%), polyuria polydipsia syndrome, with renal calcification, stones and foci of bone resorption seen on imaging (20, 21). Skeletal system pathology manifests as bone pain, osteoporosis, pathological fractures, brown tumors (rare), chondrocalcinosis (12-37% of cases), and sacroiliitis, with reduced bone mineral density present in 50-65% of patients, with the spine, hips, and distal radius being the typical sites of involvement; and a two-fold increase in the risk of nontraumatic fracture, and a three-fold increase in the risk of vertebral fracture (20, 21). Asymptomatic patients with PHPT have no obvious clinical symptoms but are found to have normal or mildly elevated blood calcium levels and elevated PTH levels by biochemical screening and may have lower than normal bone mineral density (BMD) (15, 16). The second core classification dimension relies on key serum calcium levels. The vast majority of PHPT cases are accompanied by serum total and/or ionized calcium concentrations that consistently exceed the upper limit of the normal reference range, defined as hypercalcemic PHPT. In contrast, normocalcemic primary hyperparathyroidism (NPHPT) is a specific subtype whose diagnostic criteria require repeated measurements (at least three times) of serum total and ionized calcium concentrations within the normal range, as well as persistently elevated serum PTH levels. All possible causes of secondary hyperparathyroidism, such as vitamin D deficiency, chronic kidney disease, and medications (e.g., thiazide diuretics), have been strictly excluded. In addition, specific descriptive terms have been derived for the severity and typicality of the disease presentation. Classic PHPT (classic PHPT) refers to a historically common phenotype of significant hypercalcemia with characteristic, severe target organ damage (e.g., imaging-confirmed subperiosteal bone resorption, brown tumors, history of recurrent kidney stones, or renal insufficiency). Mild PHPT (mild PHPT), on the other hand, is primarily used to describe the subset of asymptomatic PHPT with less severe biochemical abnormalities, characterized by mildly elevated calcium (often near or only slightly above the upper limit of normal) and mildly elevated PTH (14–16).

Biochemical features are the core basis for the diagnosis of PHPT, and hypercalcaemia is defined as serum total calcium >2.6 mmol/L or free calcium >1.35 mmol/L. Other etiologies such as malignancy need to be excluded, and severe hypercalcaemia (>3.5 mmol/L) can lead to acute complications (20–23). Because PTH inhibits renal tubular phosphorus reabsorption, blood phosphorus is often lower than normal in patients with PTPH, but there is no clear diagnostic threshold (21). In PHPT, a strong correlation exists between moderate hypophosphatemia (1-1.99 mg/dL) and the severity of biochemical manifestations. Historically, such isolated biochemical markers have been explored to predict surgical need in asymptomatic patients (24). However, contemporary management, as emphasized in the 2025 SFE-AFCE-SFMN Consensus, advocates for a comprehensive “target organ” approach over reliance on any single biochemical parameter for surgical decision-making in asymptomatic primary hyperparathyroidism (PHPT) (1, 25). This paradigm shift is underscored by the consensus that treatment modalities must account for expected benefits and risks, moving beyond intermediate criteria like calcemia or phosphatemia towards robust clinical endpoints such as prevention of osteoporosis or reduction in complication incidence (1). The current guidelines recommend that the indication for surgery in asymptomatic PHPT should be based on a systematic evaluation of subclinical target organ involvement, including: BMD assessment (T-score < -2.5 at lumbar spine, total hip, femoral neck, or distal 1/3 radius) (25, 26). Vertebral imaging (X-ray or VFA) to identify asymptomatic vertebral fractures (25, 26). Renal function tests and imaging to exclude nephrolithiasis/nephrocalcinosis, even in the absence of symptoms (25, 26). Furthermore, the consensus highlights specific biochemical thresholds that warrant intervention, such as serum calcium >0.25 mmol/L above the upper limit of normal (25, 26). Notably, severe hypercalcemia (≥3.5 mmol/L) constitutes an emergency requiring immediate hospitalization and rapid preoperative preparation to stabilize the patient for surgery, irrespective of symptomatic status (27). Thus, while hypophosphatemia may reflect disease activity, it is the evidence of end-organ damage or the exceedance of well-defined biochemical thresholds that now forms the cornerstone of the surgical decision-making process in asymptomatic PHPT.

Hyper PTHaemia is manifested by PTH levels that are persistently above the upper limit of normal (>65 pg/mL) and remain inappropriately normal or elevated in the presence of elevated blood calcium (28). Abnormal urinary calcium excretion reflects impaired tubular reabsorption, with hypercalciuria manifesting as >7.5 mmol/24h (300 mg/24h) in males and >6.25 mmol/24h (250 mg/24h) in females, or fasting urinary calcium concentrations >3.8 mmol/L (150 mg/L) (20, 21). Together, these biochemical markers constitute the PHPT pathophysiological network, which lays the foundation for the subsequent construction of a stratified diagnostic framework.

### 1.3 Evolving clinical spectrum: from symptomatic to asymptomatic PHPT

The clinical spectrum of primary hyperparathyroidism (PHPT) has evolved significantly over the past decade and a half. Minisola et al. demonstrated that the clinical presentation of PHPT in China is gradually shifting from a symptom-dominant to an asymptomatic phenotype, a shift that is largely attributed to the popularization of routine screening for serum calcium and the increase in the number of parathyroid lesions accidentally detected on neck ultrasonography (29). Similarly, 35 years of data from Shinshu University Hospital in Japan showed a significant increase in the proportion of asymptomatic PHPT from 51.2% to 81.8% after 2001 (P<0.01), and almost all newly diagnosed cases were of the asymptomatic form after 2016 (30). This trend is closely related to serum calcium screening in health examinations, which is widely practiced in Japan. Developing countries have similarly shown a rapid increase in asymptomatic PHPT. Data from the Indian PHPT registry (31) revealed that the proportion of asymptomatic PHPT jumped from an initial 0% to 14% between 1995–2019 and more than quadrupled between 2010–2019 compared to the previous decade (from 3% to 13%, p=0.003). A cross-national review by Arjunan et al. (32) further noted that the percentage of asymptomatic PHPT has reached 42.4%-52.5% in China, more than 58.6% in Japan, 47-81.8% in Brazil, and 24% in Eastern European countries such as Turkey and Bulgaria. Together, these findings suggest that the global clinical presentation of PHPT is converging towards asymptomatic. However, the prevalence of asymptomatic PHPT in the general population (0.18%-3.1%) may be grossly underestimated (12). Wermers (33) emphasized that a significant proportion of patients are not diagnosed in a timely manner due to the uneven implementation of serum calcium testing in primary care.A systematic evaluation by Kulkarni et al. (34) further confirmed that even if categorized as “asymptomatic”, some of the patients with PHPT still have subclinical organ damage (e.g., osteoporosis, renal structural changes) or nonspecific neurocognitive symptoms. In light of this finding, the current classification system based on biochemical markers and traditional symptoms needs to be optimized to more accurately identify high-risk populations in need of intervention. The available evidence supports the inference that the global rise in the proportion of asymptomatic PHPT is the result of a combination of advances in medical screening technology, deepening disease awareness, and population aging. However, this evolution also presents new clinical challenges - how to identify progressive cases that truly require intervention among the high proportion of asymptomatic patients will be a central theme in the future management of PHPT.

According to the 2025 consensus, asymptomatic PHPT should be classified using updated biochemical criteria and subclinical target organ damage assessment. Key parameters include: (1) serum calcium > 0.25 mmol/L (10 mg/L) above the upper normal limit; (2) eGFR < 60 mL/min/1.73m^2^ (CKD-EPI equation); (3) 24-hour urinary calcium excretion > 250 mg/d (women) or > 300 mg/d (men); (4) T-score ≤ -2.5 SD at any site or radiologically confirmed asymptomatic vertebral fractures (spinal CT/VFA recommended). Age < 50 years (or < 70 years with life expectancy adjustment) is an independent surgical indication (Grade A++) (25). The diagnostic workflow requires a multidimensional approach: (1) History & Biochemical Profiling: Repeated measurements of serum calcium, PTH, 25(OH)D, creatinine (eGFR calculation), and 24-hour urinary calcium; (2) Advanced Imaging: Spinal lateral view imaging (CT preferred over X-ray) for occult fractures, renal CT to detect asymptomatic lithiasis (11.3%) and nephrocalcinosis (10.2%); (3) Dynamic Monitoring: For non-surgical candidates, repeat eGFR, urinary calcium, and bone density every 6–12 months (25, 35).

### 1.4 Limitations of conventional diagnosis and treatment strategies

The treatment strategy for PHPT requires stratified decision-making based on lesion characteristics, patient status, and dynamic assessment of biochemical markers. Surgical treatment, as a curative tool, is indicated for symptomatic or asymptomatic patients who established surgical indications include: (1) serum calcium >0.25 mmol/L above the upper limit of normal; (2) evidence of skeletal involvement such as history of fragility fractures, radiographic signs of osteitis fibrosa cystica (e.g., subperiosteal bone resorption), or T-score ≤ -2.5 SD at any site; (3) renal complications including nephrolithiasis/nephrocalcinosis by imaging, hypercalciuria (>250 mg/24h in women or >300 mg/24h in men), or reduced creatinine clearance (<60 mL/min); and (4) age <50 years (14, 25, 26, 36). Unilateral or focused parathyroidectomy is indicated for preoperative imaging of a definite single adenoma (80-85% of cases), with a cure rate of >95%. Bilateral neck exploration is used for negative or inconsistent preoperative imaging, multi-adenomalous disease, or intraoperative parathyroid hormone (iPTH) failure (<50% decline), with a cure rate of >90% (25). Initial surgery has a high cure rate, achieving 96.6% for single adenomas and 94.5% for multiple glands. Hereditary cases (e.g. MEN1) have a high recurrence rate, with a 5-year recurrence rate of approximately 1.56% in disseminated cases (1). Complications include transient hypocalcaemia (5-52%), permanent hypoparathyroidism (<5%), recurrent laryngeal nerve injury (0.2%-1.6%) and cervical hematoma (0.3%) (25). Thermal ablation offers an alternative for patients with solitary adenomas who cannot tolerate surgery or refuse open treatment, with Microwave Ablation (MWA) and Radiofrequency Ablation (RFA) having ultrasound-guided technical success rates of >98%, with cure rates increasing to 95% for nodule diameters >0.6cm (37, 38). The complete remission rate after treatment varies widely (48%-98%) and multiple treatments are required, with Chinese studies reporting 74.7% remission rates after a single microwave ablation and 81.3% after a second (25). The recurrence rate is 2.6%-13%, and the main complications are transient recurrent laryngeal nerve palsy (5-38%), local edema (5-10%), and transient hypocalcaemia (7%) (25).

Medical therapy, primarily involving calcimimetics such as cinacalcet and antiresorptive agents like bisphosphonates, serves as an alternative for patients with PHPT who are not candidates for surgery or decline parathyroidectomy. These pharmacological options do not offer a cure but aim to manage biochemical abnormalities and reduce complications, particularly in those with contraindications to surgery or persistent disease after operation (1, 25). Cinacalcet is indicated in patients with symptomatic or marked hypercalcemia (serum calcium >0.25 mmol/L above the upper limit of normal), especially when surgery is contraindicated or refused. It normalizes serum calcium in approximately 90% of patients by allosterically modulating the calcium-sensing receptor, leading to reduced PTH secretion (39). However, its effect on PTH is partial, and no significant improvement in bone mineral density (BMD) has been demonstrated. Common side effects include nausea and hypophosphatemia, though severe adverse events are rare (1). Bisphosphonates, such as alendronate, are recommended for PHPT patients with osteoporosis (T-score ≤T-sc SD) to mitigate skeletal complications. They significantly increase BMD at the lumbar spine and hip over 1–2 years, comparable to their effects in postmenopausal osteoporosis, but show limited efficacy at the radial site. Transient reduction in serum calcium may occur shortly after initiation, yet no sustained effect on hypercalcemia or PTH levels is observed. Safety profiles are generally favorable, though caution is advised in renal impairment (1, 40). Combination therapy—using cinacalcet to address hypercalcemia and bisphosphonates or denosumab to counteract bone loss—represents an emerging tailored approach for complex cases, such as patients with both significant hypercalcemia and osteoporosis. Studies suggest that such combinations can normalize calcium and improve BMD more effectively than monotherapies, albeit with close monitoring required to avoid hypocalcemia, especially in individuals with underlying renal dysfunction or vitamin D deficiency (1). Combination regimens and supportive care allow a more personalized management approach, though periodic reevaluation remains essential to address treatment tolerance and disease progression.

The disease heterogeneity of PHPT significantly limits the generalizability of conventional diagnostic and treatment strategies. Available evidence suggests that PHPT is not only pathologically heterogeneous in terms of monoadenomas versus polyglandular lesions, but also highly differentiated in terms of clinical presentation according to genetic background (e.g., MEN1/CDC73 mutation), age (early-onset vs. senile), and calcium metabolism (typical hypercalcemic vs. normocalcemic) (1, 25, 41). However, the current diagnostic framework still faces multiple limitations: imaging techniques (e.g., ultrasound, MIBI) are insufficiently sensitive for multiglandular lesions, indications for surgery are overly dependent on blood calcium thresholds at the expense of molecular prognostic markers, and pharmacological therapies (e.g., cinacalcet) only provide symptomatic relief rather than reversal of the disease process (1, 25, 41). These limitations highlight the urgency of reconfiguring the stratified treatment system for PHPT.

Recent advances, including single-cell sequencing revealing parathyroid cellular heterogeneity and artificial intelligence-enabled integration of multimodal data (42, 43), have opened new avenues for precision medicine. Therefore, this review aims to synthesize emerging evidence on multilevel biomarkers—encompassing genetic, epigenetic, non-coding RNA, metabolic, and imaging-based markers—and to propose a novel precision medicine framework for PHPT that integrates these biomarkers into dynamic risk stratification and individualized treatment strategies. Ultimately, by combining molecular profiling, functional imaging, and real-time biomarker monitoring, we anticipate the development of an individualized decision-making framework capable of overcoming the limitations of the current “one-size-fits-all” approach, thereby advancing the paradigm from empirical management toward precision medicine in PHPT.

## 2 Evolution of biomarkers

### 2.1 Limitations of traditional markers

Available studies have demonstrated significant limitations of the traditional stratification system for PHPT based on binary judgement of blood calcium and PTH levels. It has been found that about 22% of patients with surgically confirmed PHPT exhibit normocalcemia (44), whereas 8% of patients even have both normocalcemia and normal-range PTH levels, a phenomenon that remains diagnostically blinded by the use of ionic calcium testing. Similarly, although calcium-to-phosphorus ratio (Ca/P) as a complementary index demonstrated sensitivity (90.5%) and specificity (93.2%) in PHPT screening (45), its efficacy in differentiating surgical indications in asymptomatic PHPT patients has been questioned (46). This result suggests that relying solely on biochemical parameters may not capture disease heterogeneity, especially for the identification of subclinical organ damage.

In the assessment of organ damage, there is a non-linear correlation between traditional stratification criteria and clinical outcomes. Weber et al. (47) revealed a strong correlation between parathyroid tumor calcium-sensing ability (EC50) and BMD damage. EC50, a measure of the receptor’s sensitivity to extracellular calcium, was determined ex vivo by measuring the concentration of calcium required to achieve half-maximal suppression of PTH secretion in dispersed parathyroid cells. Lumbar spine T-scores were significantly lower in patients in the high EC50 group (with reduced calcium sensitivity) than in those in the low EC50 group (- 2.7 vs -0.9), but this molecular signature was not reflected in routine blood calcium or PTH tests. This finding implies that the existing stratification system ignores the molecular heterogeneity of parathyroid function, resulting in some high-risk patients not being recognized in time. Similarly, Voss et al. (48) demonstrated that muscle function impairments such as reduced grip strength (p=0.005) and gait speed (p<0.001) were still present in patients with PHPT with normal blood calcium, and that conventional metrics failed to reflect such non-classical complications.

However, existing clinical guidelines have not systematically integrated these advances. Castellano et al. (49) further demonstrated that low blood phosphorus levels (<2.5 mg/dL) were positively correlated with PHPT severity, and that 100% of patients with moderate hypophosphatemia were eligible for surgical indication, but this metric has not been incorporated into existing stratification systems. This evidence highlights the structural shortcomings of traditional stratification tools for dynamic risk assessment and precision interventions, and there is an urgent need for biomarker-driven framework reengineering to enable optimization of therapeutic decision-making.

Existing studies have demonstrated the insidious progression of subclinical organ damage in PHPT and the lack of risk stratification tools due to the insufficient sensitivity of existing monitoring tools. Several studies have confirmed the prevalence of subclinical cardiovascular and renal damage in patients with PHPT, even when traditional surgical indications are not met. For example, early changes in renal parenchyma and vasculature in asymptomatic PHPT patients can be detected by quantitative ultrasound tools (ARFI imaging and Renal Resistance Index), with Shear Wave Velocity (SWV) and RRI values significantly correlating with blood calcium and PTH levels, suggesting that subclinical renal injuries are closely related to disease activity (50). Similarly, Aortic Intima-Media Thickness (aIMT) was significantly thickened in patients with PHPT and was independently correlated with blood and urinary calcium levels, whereas conventional carotid measurements did not show a difference, suggesting that more sensitive and targeted indices are required for the assessment of vascular injury (51). This phenomenon is also reflected at the metabolic level: PTH levels in patients with PHPT are positively correlated with insulin resistance and elevated blood pressure (52).

Long-term prognostic data gaps are particularly prominent in the field of kidney injury. A retrospective-prospective study in an Asian Indian population showed that in more than 5 years of follow-up after radical parathyroidectomy (PTX), 10.4% of 48 patients presented with eGFR <60 mL/min/1.73m^2^, 22.9% had renal tubular dysfunction including low molecular weight proteinuria and distal tubular acidosis, and the decline in eGFR was more significant in those with higher preoperative PTH levels and longer follow-up (53). This finding suggests that subclinical kidney injury may continue to progress even when biochemical parameters return to normal. However, existing guidelines have not included such subclinical kidney injury in the surgical decision criteria, resulting in some patients missing the window for early intervention (54).

The controversy over the timing of intervention stems from insufficient evidence of reversibility of subclinical damage. Randomized controlled trials have shown that PTX improves BMD and vertebral fracture risk, but the effect on non-classical symptoms (e.g. cardiovascular and neurocognitive function) is inconsistent (55). For example, improvements in postoperative anxiety and spatial working memory in patients with PHPT have been associated with a decrease in PTH, but long-term follow-up data are lacking for such neurocognitive changes (56). In light of this finding, future guidelines need to clarify thresholds for dynamic monitoring of subclinical impairments, for example, by including aortic intima-media thickness ≥1.5 mm as an extended criterion for surgical indication (sensitivity 80.6%, specificity 89.1%) (51).

Available evidence supports that accurate stratification of PHPT requires the integration of multidimensional biomarkers, including quantitative imaging parameters (aIMT, SWV), metabolic ratios (Ca/P, Ca-Cl/P) and dynamic PTH-calcium regulatory features (e.g., correlation of parathyroid tumor calcium sensitivity EC50 values with BMD damage) (47). However, there are no uniform criteria for defining progression thresholds for subclinical impairment, and factors such as vitamin D deficiency and seasonal fluctuations may interfere with marker stability (57). Therefore, reconstructing the decision-making framework requires the establishment of large-scale longitudinal cohorts to validate the causal associations between emerging markers and end-organ damage, and the development of dynamic intervention pathways based on risk stratification.

### 2.2 Breakthroughs in novel molecular markers

#### 2.2.1 Hereditary markers and driver gene mutations

Existing studies have revealed progress in the molecular mechanisms and novel biomarkers of PHPT at multiple levels. At the genetic level, the pathogenesis of familial PHPT is closely associated with mutations in several genes, including *MEN1*, *CDC73*, *CASR*, and *GCM2*, and embryonic or somatic mutations in these genes have been shown to be associated with different clinical phenotypes. In addition, other key genes such as *RET* and *CDKN1B* have been implicated in syndromic forms of PHPT. Germline mutations in *RET* are responsible for multiple endocrine neoplasia type 2A (MEN2A), in which PHPT presents alongside medullary thyroid carcinoma and pheochromocytoma (58). Similarly, mutations in *CDKN1B*, a cyclin-dependent kinase inhibitor, have been identified in patients with MEN4 syndrome, characterized by PHPT and pituitary adenomas (59). For example, functionally acquired mutations in the *GCM2* gene (e.g., p.K388E and p.V382M) showed a mutation frequency of 1.3% in the Chinese PHPT cohort and were associated with a malignant tendency to parathyroid carcinoma or atypical adenoma (60). Similarly, inactivating mutations in the *CASR* gene are not only associated with familial hypocalcemic hypercalcaemia (FHH), but may also promote disease progression through abnormalities in calcium-sensitive receptor signaling pathways in sporadic PHPT (61, 62). Notably, heterozygous versus pure mutations in the *CASR* gene exhibit different thresholds for calcium and PTH levels in neonatal severe PHPT (NSHPT), and serum calcium >4.5 mM may serve as a reliable biomarker for the diagnosis of pure mutations (61).

Beyond the well-established drivers MEN1 and CDC73, recent genomic studies have unveiled novel genetic contributors to both sporadic and hereditary PHPT, refining our understanding of its molecular substratification. Germline activating mutations in the GCM2 gene, encoding a parathyroid-specific transcription factor, have emerged as significant predisposing factors. These mutations, particularly within the C-terminal inhibitory domain (e.g., p.Y394S, p.L379Q), are enriched in patients with familial isolated hyperparathyroidism (FIHP) and are also identified in a subset of seemingly sporadic cases (63). The p.Y394S variant demonstrates a pronounced ethnic predisposition, being overrepresented in Ashkenazi Jewish populations (63). Notably, the overall frequency of activating GCM2 variants (including p.Y282D) in sporadic parathyroid adenomas is approximately 6.57%, which is about threefold higher than in the general population, suggesting a role as a moderate-risk or low-penetrance allele (63). While the penetrance of these variants is low—meaning the vast majority of carriers will not develop PHPT—their identification is crucial for recognizing familial syndromes that may present with atypical features, such as older age at onset and a lack of family history, which can mimic sporadic disease (64, 65).

Concurrently, research into tertiary hyperparathyroidism (THPT) has identified ​somatic mutations in genes like PRKDC and TBX20 that may drive malignant transformation and disease progression. Whole-exome sequencing of Chinese THPT patients revealed PRKDC, a critical gene in DNA double-strand break repair, harbored recurrent loss-of-function mutations (e.g., stop-gained in exon 21) in 5 out of 11 samples (11). These mutations were associated with downregulated mRNA expression and are postulated to contribute to uncontrolled parathyroid cell dysplasia by impairing genomic stability. Similarly, recurrent missense mutations in TBX20 (e.g., p.F282L) were found in 3 different THPT patients and were also linked to reduced gene expression, suggesting a potential damaging role in tumorigenesis (11).

The integration of these novel genetic markers (GCM2 for heritable predisposition and PRKDC/TBX20 for somatic progression) into molecular subtyping schemes offers a more granular view of PHPT pathogenesis. It enables the distinction of a subset of “sporadic” cases with an underlying genetic predisposition (GCM2-related) and identifies those at potential risk for more aggressive disease (PRKDC/TBX20-related), thereby paving the way for more personalized surveillance and management strategies.

#### 2.2.2 Epigenetic alterations: the role of DNA methylation

Epigenetic regulation, particularly aberrant promoter hypermethylation, serves as a pivotal mechanism in PHPT tumorigenesis, acting synergistically with genetic alterations (66–68). Genome-wide methylation profiling reveals *RIZ1* (PRDM2) silencing via promoter hypermethylation in 36% of sporadic parathyroid adenomas, where its loss dysregulates cell cycle by modulating histone methyltransferase activity (66). *APC* hypermethylation occurs in 75% of parathyroid carcinomas—higher than in adenomas (~71%)—activating Wnt/β-catenin signaling (66, 68). *RASSF1A* is methylated in 98% of adenomas, impairing cell cycle checkpoints (66). Promoter hypermethylation of *CDKN2A*/p16 and *CDKN2B*/p15 is considerably more common in parathyroid carcinomas than in benign adenomas, and it shows a positive correlation with increased cell proliferation as indicated by Ki-67 overexpression (66). Promoter hypermethylation of the *RB1* gene is observed in sporadic parathyroid carcinomas, representing an epigenetic mechanism for its tumor suppressor inactivation. Hypermethylation of *WT1* and *GATA4* gene promoters has been identified in parathyroid adenomas, potentially disrupting transcriptional programs involved in embryonic development. *PYCARD* (ASC/TMS1) hypermethylation is likely to inhibit its pro-apoptotic signaling and is enriched in tumors with aggressive behavior. Promoter hypermethylation of the SFRP family genes (*SFRP1*, *SFRP2*, *SFRP4*) is a frequent occurrence in parathyroid tumors. This event promotes nuclear accumulation and activation of β-catenin by relieving inhibition on the Wnt pathway (66). Complementing these findings, Zhou et al. provided direct evidence for promoter hypermethylation of the pro-opiomelanocortin (*POMC*) gene in parathyroid adenomas (69). Their integrated analysis of transcriptome and methylome data revealed *POMC* as a candidate gene exhibiting promoter hypermethylation coupled with significant downregulation at the mRNA and protein levels. Methylation-specific PCR(MSP) confirmed a higher frequency of *POMC* promoter hypermethylation in adenomas (7/10) compared to normal parathyroid tissues (4/6), suggesting this epigenetic silencing may contribute to the pathogenesis of PHPT by altering the local neuroendocrine milieu (69). These epigenetic alterations, characterized by gene-specific hypermethylation, not only elucidate the molecular pathogenesis of PHPT but also present a repertoire of potential biomarkers for refining tumor stratification and prognostication.

#### 2.2.3 Non-coding RNAs: from miRNA to lncRNA

Recent studies utilizing high-throughput technologies have systematically profiled non-coding RNAs (particularly miRNAs and lncRNAs) in parathyroid tumors, revealing their critical roles in discriminating malignant from benign lesions and assessing tumor aggressiveness. In the field of non-coding RNAs, circulating microRNA profiles showed the potential to differentiate between PHPT-associated osteoporosis and estrogen-deficient bone loss, with miR-93-5p being significantly down-regulated in the plasma of PHPT patients, while miR-24-3p was negatively correlated with lumbar spine and hip bone mineral density T-values (70). Lee et al. found a significant negative correlation between the fold change of miR-23a-5p (postoperative/preoperative expression ratio) and the change of BMD of femoral neck by analyzing serum miRNAs in 12 PHPT patients (71). A further study extended the screening of miRNAs to include 16 miRNAs known to be associated with osteoporotic fractures, and in 12 PHPT patients who underwent successful parathyroidectomy, high preoperative expression levels of miR-122-5p and miR-375 were significantly and negatively correlated with the degree of recovery of total hip (TH) BMD at 1 year postoperatively (72). Bioinformatics analysis revealed that both miRNAs co-targeted to regulate the osteogenic key transcription factor RUNX2, suggesting that it may lead to limited bone formation by inhibiting RUNX2-mediated osteogenic differentiation. In addition, it was found that patients with high preoperative miR-122-5p expression showed a smaller decrease in the bone resorption marker CTx at 2 weeks postoperatively, whereas there was no significant difference in the changes of the bone formation marker P1NP, suggesting that miR-122-5p may affect bone metabolic homeostasis by enhancing osteoclastic activity or attenuating osteogenic-osteoclastic coupling. In addition, classical markers such as blood calcium levels were positively correlated with TH BMD recovery, whereas baseline PTH was not associated with BMD changes, highlighting the advantage of miRNAs in predictive specificity (72).

Genetically, the hsa-miR-30e gene polymorphism (ss178077483 and rs7556088) showed significant differences in patients with disseminated PHPT, and the expression of this miRNA was higher in patients with multiglandular disease (MGD) than in patients with monoadenomas, which provides a new direction for molecular typing (73). However, there is no clear evidence to support its use as an independent diagnostic marker, and its specificity in familial PHPT (e.g., MEN1 or RET mutation carriers) needs to be further verified (68). Krupinova et al. (74) first reported significant downregulation of serum circulating miRNA-342-3p in PCa patients (p=0.02), with an AUC of 0.888. Its expression correlated positively with preoperative calcium and iPTH levels (r=0.52 and 0.68). A combination model integrating miRNA-342-3p, calcium, and iPTH further improved diagnostic accuracy (AUC=0.951, sensitivity 92%, specificity 91%).

Other studies consistently observed downregulation of miR-296-5p, miR-139-3p, miR-126-5p, miR-26b, and miR-30b in PCa tissues, while miR-222, miR-503, and miR-517c were upregulated. The downregulation of miR-296-5p in PC tissues has been linked to the overexpression of its target, Hepatocyte Growth Factor-Regulated Tyrosine Kinase Substrate (HGS), which promotes tumor cell invasiveness and metastasis by downregulating E-cadherin and disrupting epithelial integrity (75). Similarly, miR-126-5p, miR-26b-5p, and miR-30b-5p are significantly underexpressed in PCs compared to adenomas (75, 76). MiR-126-5p acts as a tumor suppressor by targeting genes like VEGF and KRAS, and its loss may facilitate cancer cell proliferation and invasion (75). The downregulation of miR-139-5p has also been validated as a promising diagnostic marker, often used in combination with others for improved accuracy (76).

Conversely, a subset of miRNAs is upregulated in PC and may drive oncogenic pathways. MiR-222-3p is frequently overexpressed in malignant tumors and contributes to cell cycle dysregulation by inhibiting the cyclin-dependent kinase inhibitor CDKN1B/p27 (75). MiR-517c-3p, a member of the chromosome 19 miRNA cluster (C19MC), demonstrates significant overexpression in PCs and is associated with higher serum calcium and PTH levels, as well as increased tumor weight (75). Its oncogenic role, potentially through the reactivation of embryonic signaling pathways, makes it a standout candidate for distinguishing malignancy (75). Another upregulated miRNA, miR-503-5p, has been implicated in the disease, though its specific targets in parathyroid tissue are less defined (75). The translational potential of these miRNAs extends to liquid biopsy. Studies on serum exosomes have identified miR-27a-5p as upregulated in PC patients, where it may activate the Wnt/β-catenin signaling pathway to promote tumor progression (75, 77). Notably, the C19MC miRNA cluster on chromosome 19q13.41 was aberrantly activated in PCa, potentially associated with metastatic behavior (75). These dysregulated miRNAs may promote malignant phenotypes by targeting cell cycle, apoptosis, and metabolic pathways.

However, the clinical application of these miRNA signatures faces challenges, including heterogeneity across studies, limited sample sizes due to the rarity of PC, and a lack of standardized detection protocols. Future efforts must focus on validating these markers in large, multi-center cohorts and integrating them with genetic (e.g., CDC73 status) and histopathological data to construct robust molecular diagnostic and prognostic models for precise stratification of parathyroid tumors.

Zhang et al. (78) identified 2641 lncRNAs and 2165 mRNAs differentially expressed between parathyroid carcinoma (PCa) and adenoma (PAd), with lncRNA PVT1 significantly upregulated in PCa (acting as an oncogene to promote proliferation) and lncRNA GLIS2-AS1 downregulated (potentially tumor-suppressive). Validation experiments demonstrated that PVT1 and GLIS2-AS1 yielded AUC values of 0.871 and 0.860, respectively, highlighting their diagnostic utility in distinguishing PCa from PAd (78). Additionally, dysregulated mRNAs in PCa were enriched in extracellular matrix (ECM)-receptor interaction and energy metabolism pathways, suggesting their involvement in malignant progression. Zhang et al. showed that in 57 patients with PHPT (including 11 parathyroid carcinomas and 46 parathyroid adenomas), the expression of long-chain non-coding RNA (lncRNA) scores and PVT1 expression levels were significantly higher in the parathyroid carcinoma subgroup than in the adenoma patients, and their elevation correlated with the severity of hypercalcaemia. The diagnostic efficacy of lncRNA score (AUC=0.872) in this study was superior to conventional PTH test in differentiating benign and malignant lesions within PHPT, especially in PHPT patients with combined hypercalcaemia, and its AUC was further elevated to 0.974 (sensitivity 85.71%, specificity 100%) (79). This finding suggests that lncRNA-associated markers may serve as an adjunct to identify parathyroid carcinoma within patients with PHPT, rather than a diagnostic indicator independent of PHPT.

In summary, lncRNAs and miRNAs serve as potential biomarkers for preoperative discrimination of parathyroid lesions and evaluation of tumor aggressiveness. Future studies should validate these molecules in larger cohorts and explore their therapeutic targeting potential.

#### 2.2.4 Signaling pathways and novel therapeutic targets

Beyond genetic and epigenetic alterations, dysregulation of key signaling pathways and functional proteins also contributes to PHPT pathogenesis and presents opportunities for novel biomarker and therapeutic development. In terms of signaling pathway regulation, TRPC3 channel proteins were significantly downregulated in PHPT tissues, suggesting a potential role in calcium homeostasis imbalance (80). Researchers found that Phosphatidylinositol 3-Kinase Catalytic Subunit Type 3 (PIK3C3) and Solute Carrier Family 40 Member 1 (SCF40M1) were significantly down-regulated in PHPT tissues by Mendelian Randomization (MR) and co-localization analysis (PIK3C3 and Solute Carrier Family 40 Member 1 (SLC40A1) are potential drug targets for PHPT, and increased expression of PIK3C3 was significantly associated with increased risk of PHPT, while high expression of SLC40A1 had a protective effect. Bayesian co-localization analysis confirmed that these two genes share causal variants with PHPT. Drug prediction analyses showed that potential inhibitors of PIK3C3 include emodin and celecoxib, while activators of SLC40A1 may be associated with folic acid. Multivariate MR analyses excluded confounding factors such as chronic kidney disease and blood calcium levels, and a phenome-wide association study (Phenome-Wide Association Study, PheWAS) showed that these two targets were not significantly associated with other traits, confirming their specificity (81). Together, these findings suggest that the molecular mechanism of PHPT involves multiple pathway abnormalities in calcium signaling, cell cycle regulation and epigenetic modifications, providing a theoretical basis for the development of targeted therapies (82).

#### 2.2.5 Metabolomic and inflammatory biomarkers

Aberrations in the molecular machinery governing calcium sensing often precede and precipitate the systemic metabolic imbalances observed in PHPT (47). At the forefront of this machinery is the CaSR, whose aberrant expression and function constitute a primary biomarker of disease. While inactivating mutations or polymorphisms in CaSR can disrupt calcium sensing and amplify PTH secretion, it is increasingly recognized that the receptor’s expression level itself may be a critical determinant of disease phenotype and progression. Agarwal et al. (83) systematically evaluated CaSR immunohistochemical expression in normal parathyroid tissues (from autopsies), normal rims adjacent to adenomas, and pathological tissues from PHPT patients. They demonstrated that normal parathyroid tissues exhibited strong (Her2/Neu 3+) albeit predominantly incomplete membranous staining, whereas PHPT tissues showed significantly reduced membranous expression. Notably, the decrease was most pronounced in parathyroid carcinoma, followed by adenoma, and was less marked in hyperplasia. Importantly, even histologically normal parathyroid tissue rimming adenomas already exhibited intermediate expression levels between true normal and adenoma tissues, suggesting that downregulation of CaSR expression may be an early event in tumorigenesis. Furthermore, the study revealed intra- and inter-tissue heterogeneity in CaSR expression, particularly within neoplastic tissues, and proposed that defects in CaSR trafficking from the cytoplasm to the cell surface might contribute to the reduced membranous expression observed in adenomas and carcinomas. These findings underscore that both qualitative (mutations/polymorphisms affecting function) and quantitative (reduced expression) deficits in CaSR contribute to the pathogenesis and clinical severity of PHPT.

Studies of metabolite ratios and ion balance markers have revealed the value of the Cl/P and Cl/Mg ratios for clinical applications. The Cl/P ratio was significantly different between normocalcemic and hypercalcemic PHPT (median 42.4 vs. 38.3) and correlated with reduced bone mass (84). This result was echoed by changes in otolin-1 levels, which, as a marker of calcium metabolism in the inner ear, positively correlated with serum PTH and total calcium (R²=0.53 and 0.32), suggesting that PHPT may contribute to otolith degradation through an imbalance in calcium homeostasis (85). Metabolomics analyses, on the other hand, revealed abnormal plasma levels of metabolites such as γ-glutamyl compounds, vitamin D3 derivatives, and asymmetric dimethylarginine (ADMA) in PHPT patients, suggesting potential biomarkers of oxidative stress and cardiovascular complications (86, 87). Among the inflammation-related markers, neutrophil-to-lymphocyte ratio (NLR) was positively correlated with preoperative PTH levels and significantly decreased postoperatively (2.26→1.77), reflecting a PTH-driven systemic inflammatory response (88). In addition, hs-CRP and IL-6 were significantly elevated in patients with asymptomatic PHPT and strongly correlated with PTH levels (r=0.820 and 0.787), suggesting that subclinical inflammation may precede the onset of organ damage (89). Novel inflammatory proteins such as MMP9, S100A4 and sCD14 were abnormally elevated in the serum of patients with PHPT, and some of these markers (e.g., S100A4 and sCD14) declined postoperatively, suggesting that surgery may partially reverse systemic inflammation (90).

The evolving landscape of biomarker research has further challenged the traditional stratification paradigm reliant solely on calcium and PTH levels. Emerging metabolic ratios, such as the calcium-phosphorus ratio (Ca/P) (91) and calcium-chloride/phosphorus ratio (Ca-Cl/P), have demonstrated promising discriminatory power. For instance, the Ca-Cl/P ratio achieved an ROC-AUC of 0.964 in differentiating normocalcemic PHPT from healthy controls, outperforming isolated calcium measurement (AUC=0.959) or the Ca/P ratio (AUC=0.956) (92). Similarly, the parathyroid function index (PF index = Ca × PTH/P) showed 94.6% specificity in distinguishing normocalcemic PHPT from secondary hyperparathyroidism due to vitamin D deficiency (93). Despite their diagnostic potential, these novel metabolic indices have not yet been incorporated into international clinical guidelines for the differential diagnosis of normocalcemic PHPT. Instead, current guidelines primarily emphasize the use of established functional tests. The thiazide challenge test, for example, has been proposed to differentiate renal calcium leak from autonomous parathyroid function: normalization of PTH after a short course of thiazides supports secondary hyperparathyroidism, whereas persistent elevation suggests NPHPT (94, 95). Similarly, an oral calcium loading test can help identify patients whose PTH secretion fails to suppress appropriately, further characterizing parathyroid autonomy (95). Although not yet widely standardized, these dynamic tests reflect a growing recognition of the need for functional assessment beyond static biochemical thresholds. Collectively, these advances suggest that multidimensional biomarker combinations and dynamic functional evaluations could refine risk stratification and diagnostic precision. However, prospective validation of their prognostic value and standardization of operational thresholds are necessary before integration into routine clinical practice.

In terms of complication prediction, renal injury markers KIM-1 and NGAL were significantly elevated in the urine of PHPT patients, especially in the creatinine clearance (CrCl) subgroup of 60–89 mL/min, with a 1.5-fold increase in the KIM-1/creatinine ratio compared to controls (96). These findings, together with the results that a Cl/Mg ratio ≤55 predicts the risk of kidney stones (sensitivity 82.4%, specificity 66.7%), suggest that a multidimensional combination of biomarkers may optimize risk stratification for renal complications (84). The integration of imaging histology and machine learning techniques has provided new ideas for preoperative localization, with a machine learning model (decision tree integration) based on Sestamibi-SPECT/CT data achieving 90% validation accuracy in the detection of multi-glandular disease, significantly outperforming conventional imaging methods (97). However, existing studies have not yet analyzed imaging features in conjunction with molecular markers (e.g. miRNAs or inflammatory factors), limiting their potential for accurate stratification.

#### 2.2.6 Integrating biomarkers for hereditary PHPT management

Hereditary forms of PHPT account for approximately 10-15% of all cases and encompass syndromes such as MEN1, MEN2A, MEN4, MEN5, HPT-JT, familial hypocalciuric hypercalcemia (FHH), and familial isolated hyperparathyroidism (FIHP) (98). The recognition of these genetic bases is paramount, as they dictate distinct clinical courses, management strategies, and familial screening protocols. These genetic disorders exhibit distinct clinical presentations, particularly in pediatric populations, where manifestations like rickets, short stature, and slipped capital femoral epiphysis are more prevalent due to associated vitamin D deficiency and severe hyperparathyroidism (99). A striking geographical variation is observed, with skeletal manifestations such as bone pain, fractures, and notably rickets being far more prevalent in Asian cohorts (71-86%) compared to their Western counterparts (13-34%) (99). This severe skeletal involvement is strongly linked to endemic vitamin D deficiency and inadequate dietary calcium intake in these regions, which can exacerbate the disease process (99, 100). While the profound hypophosphatemia and bone demineralization characteristic of rickets can be a consequence of severe PHPT from any cause, specific genetic mutations are associated with particularly aggressive disease. For instance, probands harboring *CDC73* mutations (causing HPT-JT syndrome) frequently present with a “severe phenotype” including renal and musculoskeletal manifestations (99). Furthermore, although less common in adolescence, biallelic *CASR* mutations are a recognized cause of neonatal severe hyperparathyroidism (NSHPT), a life-threatening disorder manifesting with severe hypercalcemia and bony deformities (98). The management of these hereditary forms is fundamentally guided by their genetics. Patients with MEN1 syndrome typically have multiglandular disease, often necessitating subtotal parathyroidectomy rather than a minimally invasive approach, and face a high risk of recurrence (99). In contrast, FHH, caused by mutations in *CASR*, *GNA11*, or *AP2S1*, is typically a benign condition that does not benefit from parathyroidectomy and must be distinguished from PHPT to avoid unnecessary surgery (98). The *CDC73* mutation carriers demand particular attention due to a substantially increased lifetime risk of parathyroid carcinoma (15-20% in adults), warranting a low threshold for en-bloc resection if malignancy is suspected and necessitating long-term vigilance (99).

Recent advances in next-generation sequencing (NGS) have highlighted the critical role of genetic screening in the clinical management of PHPT. Evidence-based guidelines, including those from the Fifth International Workshop, emphasize that genetic evaluation should be considered in specific high-risk populations to clarify etiology, guide therapy, and facilitate familial risk assessment (101, 102). The primary indications for genetic screening include early-onset PHPT (diagnosis at age <30 years), which is associated with a higher prevalence of germline mutations. For instance, a study of 107 PHPT patients undergoing NGS showed that 76.9% of those carrying pathogenic or likely pathogenic variants were under 40 years old, and met at least one high-risk criterion (103). Additionally, patients with multiglandular disease, recurrent or persistent PHPT after surgery, or those with a personal or family history suggestive of hereditary syndromes (e.g., MEN, HPT-JT, FHH) should be referred for genetic testing (101). Parathyroid carcinoma or atypical adenoma found on histopathology also warrants germline mutation analysis of genes such as *CDC73* (102, 103). Moreover, genetic testing is crucial in distinguishing FHH from PHPT in cases with mild hypercalcemia and hypocalciuria, as patients with inactivating mutations in *CASR*, *GNA11*, or *AP2S1* do not benefit from parathyroidectomy (101). In summary, targeted genetic screening in these predefined cohorts allows for precise subclassification of PHPT, informs surgical decision-making, and provides opportunities for proactive family counseling.

The key biomarkers discussed throughout this section, along with their proposed functions and clinical implications, are comprehensively summarized in **Table 1** below.

**Table 1:** Summary of novel molecular biomarkers in primary hyperparathyroidism.

Biomarker class	Specific biomarker	Main finding/function	Clinical implication/significance	Reference
Genetic	*GCM2* mutation (e.g., p.K388E, p.V382M)	Mutation frequency of 1.3% in Chinese PHPT cohort; associated with malignant tendency.	Potential predictor for parathyroid carcinoma or atypical adenoma.	(60)
Genetic	*CASR* inactivating mutations	Associated with FHH and may promote disease progression in sporadic PHPT.	Crucial for distinguishing FHH from PHPT to avoid unnecessary surgery.	(61, 62)
Genetic	Activating *GCM2* variants (e.g., p.Y394S, p.L379Q, p.Y282D)	Enriched in FIHP and sporadic cases; overall frequency ~6.57% in sporadic adenomas.	Identifies a subset of sporadic cases with underlying genetic predisposition.	(63)
Genetic	*PRKDC* mutations	Recurrent loss-of-function mutations found in THPT; associated with downregulated mRNA.	Driver of malignant transformation through impaired genomic stability.	(11)
Genetic	*TBX20* mutations (e.g., p.F282L)	Recurrent missense mutations found in THPT; linked to reduced gene expression.	Potential damaging role in tumorigenesis and disease progression.	(11)
Epigenetic	*RIZ1*/PRDM2 promoter hypermethylation	Silenced in 36% of sporadic adenomas, dysregulating cell cycle.	Potential biomarker for tumor stratification.	(66)
Epigenetic	*APC* promoter hypermethylation	Occurs in 75% of carcinomas (~71% in adenomas), activating Wnt/β-catenin signaling.	More common in carcinoma, potential marker of malignancy.	(66, 68)
Epigenetic	*RASSF1A* promoter hypermethylation	Methylated in 98% of adenomas, impairing cell cycle checkpoints.	Highly prevalent event in adenoma pathogenesis.	(66)
Epigenetic	*CDKN2A*/p16 & *CDKN2B*/p15 promoter hypermethylation	More common in carcinomas; correlates with increased cell proliferation (Ki-67).	Marker of malignant transformation and proliferation.	(66)
Epigenetic	*POMC* promoter hypermethylation	Higher frequency in adenomas (7/10) vs normal tissues (4/6); coupled with downregulation.	May contribute to pathogenesis by altering local neuroendocrine milieu.	(69)
miRNA	miR-122-5p	High pre-op expression negatively correlated with post-op BMD recovery; inhibits RUNX2.	Predictor of limited bone formation recovery after parathyroidectomy.	(72)
miRNA	miR-375	High pre-op expression negatively correlated with post-op BMD recovery.	Potential predictor of limited bone mass recovery.	(72)
miRNA	miR-342-3p	Significantly downregulated in PCa patients (p=0.02), AUC=0.888. Correlates with Ca and iPTH.	Promising diagnostic biomarker for parathyroid carcinoma.	(74)
miRNA	miR-296-5p, miR-139-3p, miR-126-5p, miR-26b, miR-30b	Downregulated in PCa tissues. miR-126-5p acts as a tumor suppressor (targets VEGF, KRAS).	Potential tumor suppressor miRNAs; loss may facilitate cancer progression.	(75, 76)
miRNA	miR-222-3p, miR-503-5p, miR-517c-3p	Upregulated in PCa. miR-222-3p inhibits CDKN1B/p27; miR-517c-3p associated with higher Ca, PTH, tumor weight.	Oncogenic miRNAs; potential markers for distinguishing malignancy.	(75)
miRNA	miR-27a-5p (serum exosomes)	Upregulated in PC patients; may activate Wnt/尾-catenin signaling.	Potential liquid biopsy marker for tumor progression.	(75, 77)
lncRNA	PVT1	Significantly upregulated in PCa vs PAd (AUC=0.871); acts as an oncogene.	Diagnostic utility in distinguishing carcinoma from adenoma.	(78)
lncRNA	GLIS2-AS1	Downregulated in PCa vs PAd (AUC=0.860); potentially tumor-suppressive.	Diagnostic utility in distinguishing carcinoma from adenoma.	(78)
lncRNA	lncRNA score & PVT1	Higher expression in carcinoma subgroup; correlates with hypercalcemia severity.	Adjunct to identify parathyroid carcinoma within PHPT patients.	(79)
Signaling Protein	TRPC3 channel proteins	Significantly downregulated in PHPT tissues.	Potential role in calcium homeostasis imbalance.	(80)
Drug Target (via MR)	PIK3C3	Increased expression associated with increased PHPT risk; potential inhibitors include emodin, celecoxib.	Potential drug target for PHPT.	(81)
Drug Target (via MR)	SLC40A1	High expression had a protective effect; activators may be associated with folic acid.	Potential drug target for PHPT.	(81)
Protein Expression	CaSR (IHC)	Reduced membranous expression in PHPT, most pronounced in carcinoma; downregulation may be an early event.	Contributes to pathogenesis and clinical severity; potential diagnostic marker.	(83)
Metabolic Ratio	Cl/P ratio	Significantly different between normo- and hypercalcemic PHPT; correlated with reduced bone mass.	Potential clinical tool for screening and assessing bone involvement.	(84)
Metabolic	Otolin-1	Positively correlated with serum PTH and total calcium.	Suggests PHPT may contribute to otolith degradation via calcium imbalance.	(85)
Metabolic	γ-glutamyl compounds, vitamin D3 derivatives, ADMA	Abnormal plasma levels in PHPT patients.	Potential biomarkers of oxidative stress and cardiovascular complications.	(86, 87)
Inflammatory	NLR (Neutrophil-to-Lymphocyte Ratio)	Positively correlated with preoperative PTH levels; significantly decreased postoperatively.	Marker for systemic inflammation in PHPT; may monitor treatment response.	(88)
Metabolic Ratio	Ca-Cl/P ratio	ROC-AUC of 0.964 in differentiating normocalcemic PHPT from healthy controls.	Superior screening tool for normocalcemic PHPT.	(92)
Metabolic Index	PF index (Ca X PTH/P)	94.6% specificity in distinguishing normocalcemic PHPT from secondary HPT due to VitD deficiency.	Aids in differential diagnosis of normocalcemic PHPT.	(93)
Kidney Injury	KIM-1, NGAL	Significantly elevated in urine, especially in CrCl 60–89 mL/min subgroup (KIM-1/Cr ratio ↑1.5-fold).	Early warning markers of subclinical kidney injury in PHPT.	(96)
Metabolic Ratio	Cl/Mg ratio (≤55)	Predicts the risk of kidney stones (sensitivity 82.4%, specificity 66.7%).	Optimizes risk stratification for renal complications.	(84)

MR, Mendelian Randomization; CrCl, Creatinine Clearance, an estimate of glomerular filtration rate (GFR) used to assess kidney function; PCa, Parathyroid Carcinoma; PAd, Parathyroid Adenoma.

## 3 Data-driven pathways for multimodal biomarker integration

### 3.1 Dynamic risk stratification models

Current research on dynamic risk stratification models for PHPT has built a full-cycle decision-making framework across disease screening, differential diagnosis and prognostic assessment by integrating multidimensional biomarkers and clinical big data. A large-scale cohort study (n=135,034) based on the TriNetX database revealed that approximately 33.3% of hypercalcaemic patients were at risk of missed diagnosis of PHPT, with the undiagnosed group with PTH ≥50 pg/mL (14.9%) and the undetected PTH group (18.4%) exhibiting a significantly differentiated comorbidity profile: the former had a 2.64-fold increase in the risk of osteoporosis over 3 years compared with matched controls, and the urinary tract had a 2.64-fold increase in the risk of osteoporosis. 2.64-fold, 2.81-fold increased risk of urinary stones, and 1.47-fold increased risk of anxiety disorders, and the symptom profile was significantly different from that of patients with diagnosed PHPT (104). Geographic differences in healthcare resources significantly affected stratification efficacy, with the rate of missed visits to healthcare facilities in the southern United States being 6 percentage points higher than in the Midwest (32.8% vs. 26.9%), suggesting that environmental factors need to be incorporated into the stratification system (104). To address the diagnostic difficulty of atypical PHPT, the machine learning model integrated seven conventional indicators, such as calcium (β=7.35), PTH (β=0.16), and vitamin D (β=0.12), through the gradient boosting algorithm, and achieved the discriminative efficacy of AUC 0.999 in 433 pathologically confirmed atypical cases, and the model successfully identified 81.6% probability of ‘threshold’ cases (105). Further time-series analysis showed that a delay in diagnosis of >1 year would result in accelerated deterioration of bone metabolism, with the incidence of osteoporosis climbing from 17.1% to 25.4% within 3 years, while delayed surgery (>1 year) increased the risk of postoperative residual hypertension by 65% (66.3% vs. 54.4%), highlighting the importance of ambulatory monitoring (104). These breakthroughs lay the foundation for a triple-play system of biomarker-clinical characteristics-medical resources for accurate stratification, but require clinical translation through multicenter external validation (especially for race-specific differences) and genomic marker integration.

Somnay et al. (106) constructed a machine learning model based on clinical and laboratory characteristics based on multicenter data (6,777 PHPT patients vs. 5,053 thyroid surgery controls) from three high volume endocrine surgery centers from 2001-2013. Core data included age, gender, preoperative calcium, phosphate, PTH, vitamin D, and creatinine levels. After testing more than 20 algorithms, including BayesNet, logistic regression, and decision tree, through the Weka platform, BayesNet demonstrated the highest accuracy in 10-fold cross-validation (overall accuracy 95.2%, AUC=0.989). For mild PHPT, the underlying Bayesian network had a classification accuracy of 71.1% for patients with normal blood calcium and 92.1% for those with normal PTH. Enhanced by the introduction of the AdaBoost meta-algorithm, the overall accuracy was improved to 97.2% (AUC=0.994), with the accuracy rate for mild cases increasing from 86.0% to 91.9% and the false-negative rate decreasing from 14% to 8.1%. Notably, the model maintained 95.6% accuracy (AUC=0.985) even when the PTH indicator was excluded, suggesting its potential application to scenarios where PTH is not routinely detected in primary care. Greer et al. (107) attempted to construct predictive models without relying on calcium and PTH data using University of Arkansas Medical Centre electronic medical record data from 2014-2019 (1,737 patients containing 185,000 records). Key risk factors were identified by feature screening as diastolic blood pressure, age, BMI, weight, ethnicity, smoking, diabetes mellitus, hypertension, renal disease, proton pump inhibitor and bisphosphonate use. An AUC of 0.86 (sensitivity 89.53%, specificity 66.86%) was achieved using a Gradient Boosting Machine (GBM) model after exclusion of calcium/PTH data. The results showed that cardiovascular-related indicators (diastolic blood pressure, diabetes mellitus, hypertension, nephropathy, smoking) and metabolism-related indicators (BMI, body weight) contributed significantly to the prediction of PHPT, suggesting a potential association of PHPT with metabolic syndrome and cardiovascular risk. Axelsson et al. (108) constructed a dynamic risk stratification model for PHPT patients by integrating multidimensional clinical data and longitudinal follow-up information. The study included 16,374 PHPT inpatients and 163,740 demographically matched controls during 2006-2017, with a mean follow-up of 1.15 years (PHPT group) and 4.62 years (control group), respectively. The study used a Cox proportional risk model combined with time-dependent Poisson regression to reveal the characteristics of risk evolution for different endpoints over the natural course of PHPT. The dynamic stratification model developed in this study comprised three core dimensions: (1) baseline risk characteristics: age, gender, Charlson comorbidity index, and history of previous fracture constituted the underlying risk stratum; (2) disease-specific indices: history of renal stone and diagnosis of osteoporosis significantly enhanced the predictive validity; and (3) response to intervention factors: the time of PTX implementation and changes in postoperative biochemical parameters constituted the dynamic adjustment stratum. In particular, the investigators emphasized that the model needs to be dynamically calibrated in conjunction with ongoing monitoring data (e.g., postoperative blood calcium, PTH levels), and that its predictive efficacy has been validated in 42,310 person-years of follow-up data from the PHPT cohort.

Beyond predicting sporadic PHPT, recent advancements in machine learning and big data analytics have also facilitated the development of models to stratify hereditary PHPT itself, particularly in distinguishing MEN1-related disease from its phenocopies. Trukhina et al. (109) employed multiple machine learning algorithms, including k-nearest neighbors (kNN), logistic regression, and random forest, to differentiate genetically confirmed MEN1 syndrome from its phenocopies based on easily accessible clinical features. Their study demonstrated that the kNN algorithm achieved exceptional performance, with a sensitivity of 94.4% and specificity of 100% in the test cohort, utilizing predictors such as the number of affected parathyroid glands, age at diagnosis, presence of pancreatic tumors, hereditary background, pituitary adenoma secretion type, and gender (109). Complementing this, Mokrysheva et al. (110) developed a clinical prediction model using logistic regression to estimate the probability of MEN1 gene mutations in young PHPT patients. Their model incorporated eight key predictors: multiglandular involvement, positive family history, PHPT recurrence, age at onset, presence of pancreatic neuroendocrine tumors, pituitary adenomas, histopathological features of parathyroid lesions, and postoperative hypoparathyroidism. This model exhibited high diagnostic accuracy, with a sensitivity of 96% and specificity of 98%, and an AUC of 0.983, underscoring its potential for optimizing genetic testing referrals and improving diagnostic precision in clinical settings (110). These studies collectively highlight the utility of integrating machine learning and statistical modeling with routine clinical parameters to enhance the stratification of MEN1-related PHPT, thereby aiding in personalized diagnostic and therapeutic decisions.

### 3.2 Advancements in multimodal imaging for precise localization and complication assessment

Modern imaging technology has evolved from purely anatomical localization into a precision tool that provides integrated functional and anatomical information, playing a pivotal role throughout the management of PHPT. This is particularly evident in two critical aspects: precise preoperative localization and differential diagnosis of pathological glands, and sophisticated assessment of end-organ complications such as skeletal involvement. This section will first discuss the advances in cutting-edge imaging techniques for preoperative localization.

#### 3.2.1 Preoperative localization and differential diagnosis

Accurate preoperative localization of hyperfunctioning parathyroid tissue is paramount in the management of PHPT, as it guides minimally invasive parathyroidectomy, reduces surgical complications, and enhances curative outcomes. Conventional imaging techniques, such as neck ultrasonography (US) and technetium-99m sestamibi (MIBI) scintigraphy, have long served as first-line modalities due to their widespread availability and cost-effectiveness. However, emerging advanced technologies, including SPECT/CT, 4D-CT, and novel PET/CT tracers like 18F-fluorocholine (FCH), offer improved diagnostic accuracy, though with trade-offs in cost, radiation exposure, and accessibility. This section discusses the comparative advantages and limitations of these modalities and explores integrated strategies for optimal preoperative planning, ultimately supporting treatment stratification by tailoring diagnostic pathways to individual patient profiles.

Ultrasonography remains a cornerstone in PHPT localization due to its non-invasive nature, absence of ionizing radiation, and real-time imaging capabilities. Studies consistently report variable sensitivity, ranging from 49.3% to 93.0% on a per-lesion basis, where US demonstrated 93.0% sensitivity compared to 63.0% for MIBI SPECT/CT in detecting parathyroid adenomas (111). US excels in characterizing gland morphology and vascularity through features like the “polar vascular sign” and “residual parathyroid sign,” particularly for larger lesions, but its accuracy is operator-dependent and diminishes for ectopic glands, multiglandular disease, or in the presence of thyroid nodules (111, 112). For instance, Chiu et al. (112) highlights that US sensitivity drops significantly in multigland disease, emphasizing its limitation in comprehensive localization.

MIBI scintigraphy, often augmented with SPECT/CT, provides functional assessment by exploiting mitochondrial uptake in hyperfunctioning parathyroid cells. While it is an established standard, its sensitivity is inconsistent, ranging from 49.3% to 63.0% (111), and it can yield false positives due to thyroid pathology or other hypermetabolic conditions. Yang et al. (113) further supports that MIBI SPECT/CT alone achieves an accuracy of 92% in per-lesion analysis, but it underperforms in multigland disease and small adenomas (<500 mg) (111). The integration of SPECT/CT improves anatomical correlation, yet it still falls short in sensitivity compared to newer modalities.

Advanced cross-sectional imaging techniques, such as 4D-CT, offer high spatial resolution and multiphase contrast enhancement patterns, achieving sensitivities up to 81-92% for single-gland disease (113). However, 4D-CT involves substantial radiation exposure, which is a significant drawback, particularly for younger patients. In contrast, 4D-dynamic contrast-enhanced MRI (4D-DCE MRI) emerges as a promising alternative, providing comparable accuracy without ionizing radiation. Becker et al. (114) reports that 4D-DCE MRI correctly localized 92% of single-gland diseases and 74% of multigland diseases, with excellent interobserver agreement (κ=0.92 for side identification), making it suitable for radiation-sensitive populations.

The advent of metabolic imaging with 18F-fluorocholine PET/CT represents a paradigm shift, leveraging choline uptake in proliferating parathyroid cells. Mathey et al. (115) and Chiu et al. (112) demonstrate its superior sensitivity (87.0% to 93.7%) and specificity (100%) compared to US and MIBI. For example, Chiu et al. (112) shows that FCH-PET/CT detected 87.0% of lesions versus 49.3% for US and MIBI individually, and it effectively localized lesions missed by conventional methods, with 80.9% sensitivity in cases with negative or discordant US/MIBI results. Additionally, Mathey et al. (115) highlights that FCH-PET/CT outperformed 11C-methionine PET/CT, with 96% per-patient sensitivity, facilitating minimally invasive surgery in 92% of cases. Despite its high cost and limited availability, FCH-PET/CT is particularly valuable for reoperative cases, multigland disease, and ectopic glands.

Integration of these modalities is key to optimizing preoperative localization. Chiu et al. (112) advocates for an ultrasound-first approach, followed by FCH-PET/CT in uncertain cases, as the combination achieved 94.2% sensitivity and 98.9% specificity, surpassing other strategies. Similarly, Lu et al. (111) suggests that combining US with MIBI SPECT/CT improves overall accuracy (88.0% for US alone vs. 97.0% for combination in some cohorts), but document 5 notes that adding 4D-CT to MIBI SPECT/CT does not significantly enhance performance, indicating careful selection is needed. For differential diagnosis, techniques like PTH washout from fine-needle aspiration (116) and immunohistochemical markers (e.g., GATA3) complement imaging by confirming parathyroid origin in equivocal cases.

In conclusion, the choice of imaging modality should be stratified based on clinical context, gland characteristics, and resource availability. While US and MIBI serve as foundational tools, advanced techniques like 4D-CT, 4D-DCE MRI, and FCH-PET/CT provide critical enhancements for complex cases. An integrated, stepwise approach—starting with US and escalating to FCH-PET/CT or MRI when needed—ensures precise localization, supports individualized treatment plans, and ultimately improves surgical outcomes, aligning with the principles of precision medicine in PHPT management.

#### 3.2.2 Advanced assessment of skeletal complications

Moving beyond the traditional assessment of BMD by Dual-Energy X-ray Absorptiometry (DXA), advanced imaging techniques now enable a more refined quantitative evaluation of bone microarchitecture for fracture risk prediction. The integration of radiomic analysis with machine learning is paving new avenues in PHPT bone assessment. For instance, a study utilizing routine CT scans demonstrated that while Hounsfield unit (HU) values had limited efficacy in differentiating osteoporosis from osteopenia, machine learning models incorporating advanced texture features (e.g., Kurtosis, grey level inhomogeneity) significantly improved classification performance (AUC: 0.77 vs. 0.65) (117). These texture parameters reflect heterogeneity in trabecular microstructure and density distribution, capturing microscopic bone degradation that may serve as novel biomarkers for predicting fracture risk and informing clinical decisions regarding surgery or medical therapy (117).

Complementing these analytical approaches, dedicated high-resolution modalities have been developed to provide direct, three-dimensional assessment of bone microstructure. High-resolution peripheral quantitative computed tomography (HR-pQCT) has emerged as a powerful modality for three-dimensional assessment of bone microstructure. Wang et al. (118) demonstrated that both cortical and trabecular compartments are significantly impaired in PHPT patients, with reduced volumetric BMD (vBMD) at both radius and tibia sites. Their study revealed thinner cortices, more widely spaced trabeculae, and decreased trabecular number in Chinese PHPT patients compared to controls, with similar abnormalities observed in both sporadic and MEN1-related PHPT cases. These microstructural alterations persisted even after adjusting for age and sex, indicating that HR-pQCT can detect skeletal deterioration that might be overlooked by DXA alone.

The development of 3D-DXA technology represents another significant advancement, allowing for separate evaluation of cortical and trabecular bone using standard DXA images. Guerra et al. (119) applied this technique in normocalcemic PHPT patients and found significant impairments in cortical parameters, particularly cortical vBMD and surface BMD (sBMD), while trabecular vBMD showed no significant differences compared to controls. Their 3D mapping analysis revealed uniformly lower cortical vBMD throughout the proximal femur, with more localized deficiencies in the intertrochanteric area, potentially explaining the increased fracture risk in this region. This regional analysis capability provides clinicians with anatomical-specific information that could guide targeted interventions.

For trabecular bone assessment specifically, the trabecular bone score (TBS) has proven valuable as a texture parameter derived from lumbar spine DXA images. Song et al. (120) conducted a comparative study between MEN1-related PHPT (MHPT) and sporadic PHPT (SHPT) patients, revealing that although the proportion of skeletal involvement was similar between groups, TBS was significantly lower in the MHPT group (1.22 ± 0.14 vs. 1.29 ± 0.11, P < 0.001). Notably, among MHPT patients with normal BMD, 44.1% had degraded bone microstructure (TBS < 1.230) and 17.6% had partially degraded microstructure, indicating that TBS can identify microarchitectural deterioration even when aBMD appears preserved. This enhanced sensitivity makes TBS particularly valuable for early detection of skeletal involvement in PHPT patients who might otherwise be considered low-risk based on DXA results alone.

In addition to the above technologies, Radiofrequency Echographic Multi Spectrometry (REMS), as an emerging radiation-free ultrasound technology, provides a new perspective for bone assessment in PHPT patients. The advantage of REMS over traditional DXA is that it completely avoids ionizing radiation and can analyze backscattered ultrasound radiofrequency signals to assess BMD at axial sites (lumbar spine and hip) while simultaneously assessing BMD. It provides a unique assessment of the quality of bone microstructure, namely Fragility Score (FS) (121). REMS has been validated for equivalence with quality-assured DXA scans in a number of studies, showing more than 90% sensitivity and specificity in the diagnosis of osteoporosis (121). More importantly, the FS provided by it combined with T-score can significantly improve the accuracy of predicting the risk of major osteoporotic and hip fractures within 5 years, and its predictive efficacy is better than that of BMD alone (121). This is of great value for the long-term follow-up and fracture risk stratification of PHPT patients, especially for special populations who need to avoid radiation exposure (such as young patients and pregnant women) or PHPT patients whose DXA assessment is limited due to severe spinal deformity, osteoarthritis, and implants (121). Although the REMS research for PHPT population is still in its infancy, its technical characteristics are highly consistent with the clinical management needs of PHPT. Future research should focus on exploring the applicability of the REMS and its FS in PHPT patients, and verify whether it can be used as a sensitive tool to predict the risk of PHPT-specific fracture and monitor the treatment effect (such as postoperative bone recovery or drug response).

The clinical implications of these advanced imaging techniques extend beyond mere detection of microstructural changes. Eremkina et al. (122) demonstrated that 3D-DXA parameters could differentiate between MHPT and SHPT patients, with MHPT showing more severe preoperative cortical bone damage despite similar biochemical profiles. Their longitudinal assessment further revealed that both groups showed significant improvement in cortical and trabecular parameters following parathyroidectomy, suggesting that these advanced imaging modalities can also monitor treatment response more comprehensively than conventional DXA.

In conclusion, the integration of HR-pQCT, 3D-DXA, and TBS into the diagnostic workflow provides a multidimensional assessment of skeletal health in PHPT patients. These technologies enable quantification of both cortical and trabecular compartment deterioration, offer regional specificity in identifying high-risk anatomical sites, and detect microarchitectural changes before they manifest as reduced aBMD. This comprehensive evaluation significantly enhances fracture risk stratification, allowing for more personalized management decisions and timely interventions to prevent skeletal complications in PHPT patients.

The key technologies and their clinical applications, as discussed in sections 3.1 and 3.2, are comprehensively summarized in **Table 2** below.

**Table 2:** Summary of advanced data-driven and imaging techniques for PHPT management.

Technology/model type	Core function/application	Key finding/performance	Clinical implication/advantage	Reference
Machine Learning (BayesNet + AdaBoost)	Differentiating PHPT from controls using clinical/lab data	Overall accuracy 97.2% (AUC=0.994); 95.6% accuracy even without PTH data.	Potential for screening in primary care where PTH is not routinely available.	(106)
Machine Learning (Gradient Boosting Machine - GBM)	Predicting PHPT without relying on Ca/PTH data	AUC 0.86 (sensitivity 89.53%, specificity 66.86%) using clinical features (e.g., BP, BMI, comorbidities).	Useful for risk stratification when classic biochemical data is missing.	(107)
Dynamic Risk Model (Cox/Poisson regression)	Long-term, dynamic risk stratification of PHPT complications	Model integrated baseline risks, disease indices, and post-op response; validated on 42,310 person-years of data.	Moves beyond static assessment to a dynamic model that evolves with patient status.	(108)
Machine Learning (k-Nearest Neighbors - kNN)	Differentiating MEN1 from phenocopies	Sensitivity 94.4%, specificity 100% using clinical features (e.g., number of glands, pancreatic tumors).	Aids in the difficult clinical differentiation of genetic syndromes without initial genetic testing.	(109)
Clinical Prediction Model (Logistic Regression)	Predicting MEN1 mutation probability in young PHPT pts	Sensitivity 96%, specificity 98% (AUC=0.983) using 8 clinical predictors.	Optimizes genetic testing referrals by identifying young patients with high pre-test probability for MEN1.	(110)
Ultrasonography (US)	Preoperative adenoma localization	93.0% sensitivity per-lesion (vs 63.0% for MIBI SPECT/CT).	Highly effective first-line, non-ionizing imaging tool, though operator-dependent.	(111)
18F-Fluorocholine PET/CT (FCH-PET/CT)	Preoperative localization, especially after inconclusive US/MIBI	87.0% detection rate; 80.9% sensitive in US/MIBI negative or discordant cases.	Superior sensitivity for challenging cases (reoperation, multigland disease, ectopic glands).	(112)
4D-CT	Preoperative localization	81-92% sensitivity for single-gland disease.	Provides excellent anatomical detail but involves substantial radiation exposure.	(113)
4D-Dynamic Contrast-Enhanced MRI (4D-DCE MRI)	Preoperative localization	Correctly localized 92% of single-gland disease, 74% of multigland disease (魏=0.92 for side ID).	Provides high accuracy without ionizing radiation, ideal for radiation-sensitive populations.	(114)
18F-Fluorocholine PET/CT (FCH-PET/CT)	Comparing PET tracers for localization	96% per-patient sensitivity; outperformed 11C-methionine PET/CT.	Highly sensitive for guiding minimally invasive surgery.	(115)
Radiomics/Machine Learning on CT	Differentiating osteoporosis from osteopenia	Texture feature model AUC: 0.77 (vs. AUC 0.65 for Hounsfield units alone).	Extracts additional bone quality data from routine CT scans for better fracture risk assessment.	(117)
High-Resolution peripheral QCT (HR-pQCT)	Assessing bone microarchitecture	Quantified cortical & trabecular deterioration (thinner cortices, fewer trabeculae) in PHPT vs controls.	Detects microstructural bone damage that is missed by standard DXA BMD measurement.	(118)
3D-DXA	Assessing bone density and microstructure	Found significant cortical vBMD impairments in normocalcemic PHPT.	Provides more detailed bone analysis from standard DXA images, separating cortical/trabecular compartments.	(119)
Trabecular Bone Score (TBS)	Assessing bone quality from spine DXA	TBS was significantly lower in MEN1-related PHPT despite similar BMD.	Identifies degraded bone microstructure in patients who may have “normal” BMD, indicating higher fracture risk.	(120)
Radiofrequency Echographic Multi Spectrometry (REMS)	Radiation-free BMD and bone quality assessment	>90% sensitivity/specificity vs. DXA; provides a Fragility Score (FS) to improve fracture prediction.	Ideal for patients where DXA is limited (e.g., young pts, pregnancy, severe scoliosis) or to avoid radiation.	(121)
3D-DXA	Differentiating bone phenotypes	Showed more severe cortical damage in MEN1-related vs. sporadic PHPT pre-op; monitored post-op improvement.	Useful for stratifying skeletal risk and monitoring treatment response beyond areal BMD.	(122)

### 3.3 Proposed precision stratification framework

**Figure 1:**
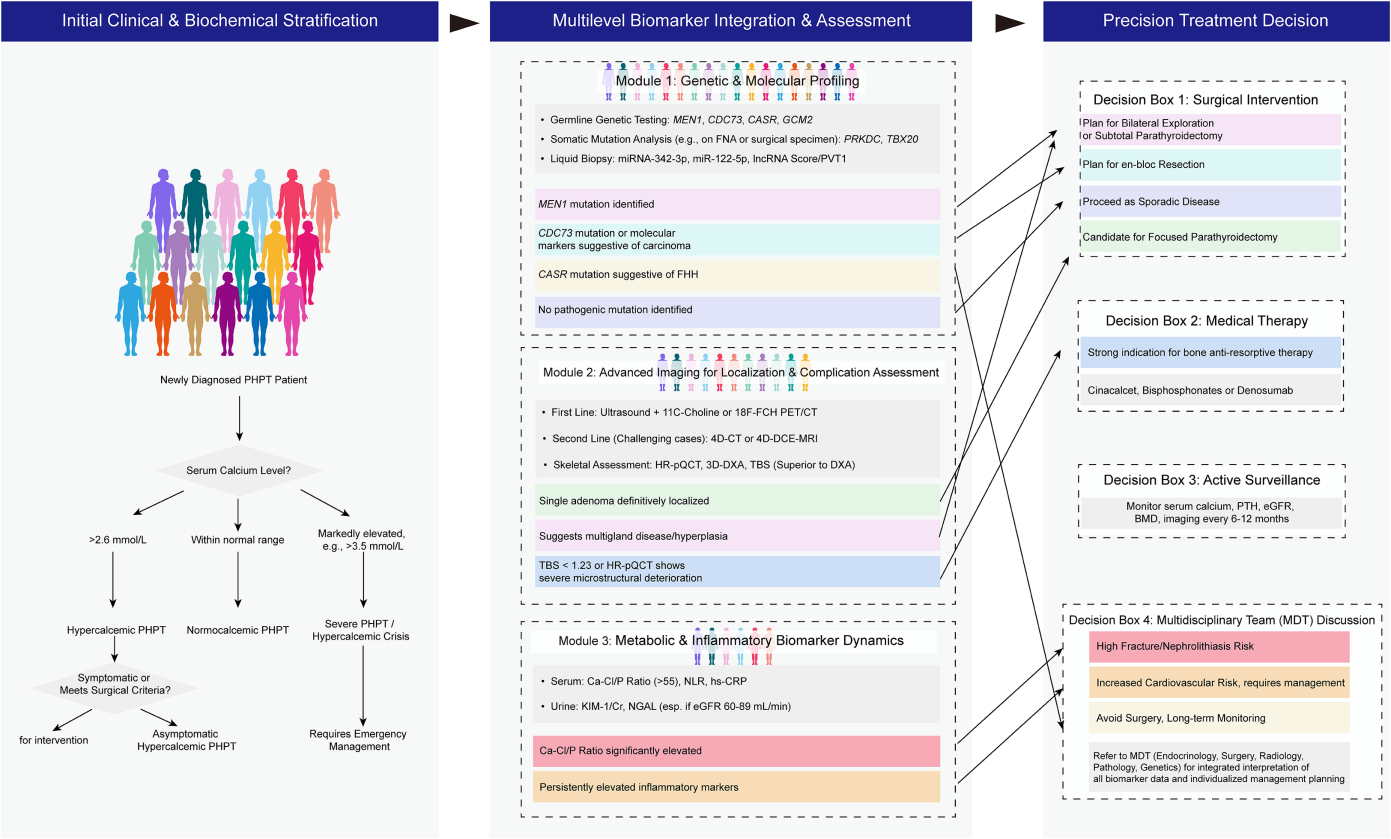
A novel evidence-based diagnostic-therapeutic algorithm integrating multilevel biomarkers to guide precision management of PHPT.

We propose a novel evidence-based diagnostic-therapeutic algorithm integrating multilevel biomarkers to guide precision management of PHPT (**Figure 1**). This framework transitions from traditional symptom- and calcium-centric approaches toward a dynamic, biomarker-stratified paradigm, aligning with the core principles of precision medicine. The algorithm is structured into three synergistic tiers: risk stratification, multimodal biomarker integration, and individualized intervention.

The initial tier employs clinical and biochemical profiling—including serum calcium, PTH, urinary calcium excretion, and bone mineral density—to categorize patients into classical hypercalcemic, normocalcemic, or high-risk symptomatic subgroups. This foundational layer triggers subsequent advanced evaluation rather than dictating final treatment decisions.

The second tier constitutes the core innovation: multidimensional biomarker integration. Genetic profiling (e.g., *MEN1*, *CDC73*, *GCM2* mutations) identifies hereditary syndromes and predicts tumor behavior, directing surgical extent and familial screening. Molecular markers (e.g., miR-122-5p, lncRNA PVT1) offer insights into bone metabolic activity and malignant potential, while metabolic ratios (Ca-Cl/P) and inflammatory indices (NLR) refine risk prediction for skeletal and renal complications. Advanced imaging-including ^18^F-fluorocholine PET/CT for localization and HR-pQCT/TBS for microstructural bone assessment-provides anatomical and functional correlation, overcoming limitations of conventional techniques.

The final tier translates these layered inputs into precision interventions. Surgical strategies are tailored to biomarker-defined risks: focused parathyroidectomy for localized adenomas, subtotal resection for multigland disease (e.g., MEN1 carriers), or en-bloc resection for carcinoma-predictive signatures (*CDC73* mutations). Medical therapy is selectively deployed—calcimimetics for hypercalcemia in non-surgical candidates, antiresorptives for high fracture risk (TBS <1.23, degraded microarchitecture)—while active surveillance is reserved for biomarker-negative, low-risk cases. Complex scenarios trigger multidisciplinary review, ensuring holistic decision-making.

This framework transcends the unidimensionality of current guidelines by incorporating temporal dynamics and validating predictions through machine learning models integrating clinical, genomic, and imaging data. It ultimately empowers a shift from reactive disease management to proactive health preservation, leveraging biomarkers not merely for diagnosis but for forecasting disease trajectory and therapeutic response.

## 4 Multidisciplinary team approach in precision management of PHPT

The complexity and heterogeneity of PHPT necessitate a collaborative, multidisciplinary approach to ensure precise diagnosis, risk stratification, and personalized treatment. An ideal MDT for PHPT should integrate expertise from endocrinology, parathyroid surgery, radiology, nuclear medicine, pathology, genetics, and data science, each contributing uniquely to the interpretation and application of multilevel biomarkers across the patient journey (123, 124).

The endocrinologist serves as the primary coordinator, initiating biochemical profiling (e.g., serum calcium, PTH, vitamin D, urinary calcium) and identifying candidates for MDT referral based on atypical presentations, normocalcemic PHPT, or suspected hereditary syndromes (123). The radiologist and nuclear medicine specialist leverage advanced imaging (ultrasonography, 99mTc-sestamibi SPECT/CT, 18F-fluorocholine PET/CT) to localize abnormal glands and assess complications like bone microarchitectural deterioration using high-resolution techniques (e.g., HR-pQCT, TBS) (125). The pathologist confirms tissue diagnosis via histopathology and immunohistochemistry (e.g., GATA3 staining) and identifies malignant features in resected specimens, while the genetic counselor evaluates germline mutations in young-onset or familial cases, facilitating risk stratification and family screening (124, 126). The parathyroid surgeon synthesizes these inputs to determine the extent of resection—focused parathyroidectomy for localized adenomas versus subtotal resection for multiglandular disease (e.g., MEN1 carriers)—aided by intraoperative PTH monitoring to confirm cure (125). Data scientists or bioinformaticians integrate multimodal data (clinical, genomic, imaging) into machine learning models to predict disease behavior, surgical outcomes, and long-term complications.

A typical patient pathway begins with primary care identifying hypercalcemia, triggering endocrine referral for comprehensive biomarker assessment. Upon MDT enrollment, the radiologist and nuclear medicine specialist perform targeted imaging, while genetic testing is initiated for high-risk cases. The MDT convenes to review data, discordances, and biomarker-based risks, culminating in a consensus on surgery, medical management, or active surveillance. Postoperatively, the endocrinologist and radiologist monitor for recurrence or complications using dynamic biomarkers.

However, the efficacy of MDT collaboration is hampered by data siloing: disparate data formats from biochemistry (e.g., Ca-Cl/P ratios), imaging (DICOM files), genomics (FASTQ sequences), and pathology (slide images) impede seamless integration. This fragmentation underscores the urgent need for unified platforms capable of harmonizing multimodal data, enabling real-time biomarker analytics and personalized decision-support tools—a gap that will be addressed in the conclusion proposing integrated informatics solutions.

## 5 Conclusion and future perspectives

This review summarizes the latest advances in precision stratified treatment of PHPT based on multilevel emerging biomarkers, encompassing breakthroughs in molecular mechanisms (e.g., genetic mutations in *MEN1*, *CDC73*, and *CASR*; non-coding RNAs like miR-122-5p and lncRNA PVT1; metabolomic ratios such as Ca-Cl/P), imaging histology for bone microstructural assessment, and artificial intelligence-driven multimodal data integration (e.g., Bayesian networks and machine learning models). These advancements have facilitated a paradigm shift from a one-size-fits-all approach to dynamic risk stratification in PHPT. However, clinical translation remains hindered by challenges such as the lack of standardized validation for novel biomarkers and the absence of integrated platforms for multi-omics data, limiting the generalizability and implementation of these strategies in routine practice.

To address these limitations, we propose the development of an AI-assisted multi-omics data sharing and clinical decision support platform, which would integrate heterogeneous data modalities (biochemical, genomic, imaging, and pathological) into a unified framework for real-time biomarker analytics and personalized risk prediction. This platform would enable the implementation of a holistic, full-cycle health management model for PHPT, spanning risk prediction, early diagnosis, precision intervention (e.g., MDT-guided surgery or medical therapy), and long-term follow-up, with MDTs central to each stage. Crucially, establishing a cross-institutional interdisciplinary collaborative network is essential for large-scale validation through multicenter prospective cohorts, fostering the creation of evidence-based, consensus-driven guidelines for biomarker application. This approach will not only bridge the gap between research and clinical practice but also pave the way for a proactive, health-prognostic-oriented paradigm in PHPT management.
